# The Expression Profiles and Deregulation of UDP-Glycosyltransferase (*UGT*) Genes in Human Cancers and Their Association with Clinical Outcomes

**DOI:** 10.3390/cancers13174491

**Published:** 2021-09-06

**Authors:** Dong Gui Hu, Shashikanth Marri, Peter I. Mackenzie, Julie-Ann Hulin, Ross A. McKinnon, Robyn Meech

**Affiliations:** 1 Dicipline of Clinical Pharmacology, College of Medicine and Public Health, Flinders University, Bedford Park, SA 5042, Australia; peter.mackenzie@flinders.edu.au (P.I.M.); julieann.hulin@flinders.edu.au (J.-A.H.); ross.mckinnon@flinders.edu.au (R.A.M.); robyn.meech@flinders.edu.au (R.M.); 2 Dicipline of Molecular Medicine and Pathology, College of Medicine and Public Health, Flinders University, Bedford Park, SA 5042, Australia; shashikanth.marri@flinders.edu.au

**Keywords:** UDP-glycosyltransferase, UDP-glucuronosyltransferase, cancer, drug metabolism, gene expression, differential gene expression, overall survival, prognostic biomarkers

## Abstract

**Simple Summary:**

The human UDP-glycosyltransferase (UGT) superfamily plays a critical role in the metabolism of numerous endogenous and exogenous small lipophilic compounds, including carcinogens, drugs, and bioactive molecules with pro- or anti-cancer activity. Previous studies have documented the expression of *UGT* genes in several cancers derived from drug-metabolizing organs (e.g., liver, colon, kidney). The present study represents the first to comprehensively assess the expression profiles of *UGT* genes and their impact on patient survival in nearly 30 different cancers primarily derived from non-drug-metabolizing organs. Briefly, our comprehensive analysis of the transcriptomic (RNAseq) and clinical datasets of 9514 patients from 33 different cancers shows the widespread expression of *UGT* genes, indicative of active drug metabolism within the tumor through the UGT conjugation pathway. We further identified the *UGT* genes whose intratumoral expression was associated with patient survival, highlighting the potential of *UGT* genes as prognostic biomarkers and therapeutic targets in various cancers.

**Abstract:**

The human UDP-glycosyltransferase (UGTs) superfamily has 22 functional enzymes that play a critical role in the metabolism of small lipophilic compounds, including carcinogens, drugs, steroids, lipids, fatty acids, and bile acids. The expression profiles of *UGT* genes in human cancers and their impact on cancer patient survival remains to be systematically investigated. In the present study, a comprehensive analysis of the RNAseq and clinical datasets of 9514 patients from 33 different TCGA (the Genome Cancer Atlas) cancers demonstrated cancer-specific UGT expression profiles with high interindividual variability among and within individual cancers. Notably, cancers derived from drug metabolizing tissues (liver, kidney, gut, pancreas) expressed the largest number of *UGT* genes (COAD, KIRC, KIRP, LIHC, PAAD); six *UGT* genes (*1A6*, *1A9*, *1A10*, *2A3*, *2B7*, *UGT8*) showed high expression in five or more different cancers. Kaplan–Meier plots and logrank tests revealed that six *UGT* genes were significantly associated with increased overall survival (OS) rates [*UGT1A1* (LUSC), *UGT1A6* (ACC), *UGT1A7* (ACC), *UGT2A3* (KIRC), *UGT2B15* (BLCA, SKCM)] or decreased OS rates [*UGT2B15* (LGG), *UGT8* (UVM)] in specific cancers. Finally, differential expression analysis of 611 patients from 12 TCGA cancers identified 16 *UGT* genes (*1A1*, *1A3*, *1A6*, *1A7*, *1A8*, *1A9*, *1A10*, *2A1*, *2A3*, *2B4*, *2B7*, *2B11*, *2B15*, *3A1*, *3A2*, *UGT8*) that were up/downregulated in at least one cancer relative to normal tissues. In conclusion, our data show widespread expression of *UGT* genes in cancers, highlighting the capacity for intratumoural drug metabolism through the UGT conjugation pathway. The data also suggests the potentials for specific *UGT* genes to serve as prognostic biomarkers or therapeutic targets in cancers.

## 1. Introduction

The human UDP-glycosyltransferase (*UGT*) superfamily contains 22 functional genes that are divided into four subfamilies (*UGT1*, *UGT2*, *UGT3*, *UGT8*) [1,2]. UGTs conjugate numerous small lipophilic endogenous and exogenous compounds at functional groups (e.g., hydroxyl, carboxyl, amine) with sugars (e.g., glucuronic acid, glucose, xylose, N-acetylglucosamine, galactose), and the resultant products are generally inactive and water-soluble, thus eliminating the biological activity of the parent compounds and facilitating their excretion from the body through the bile, urine or feces [3]. The 9 UGT1 (1A1, 1A3-1A10) and 10 UGT2 (2A1, 2A2, 2A3, 2B4, 2B7, 2B10, 2B11, 2B15, 2B17, and 2B28) enzymes conjugate substrates with glucuronic acid and are hence traditionally termed UDP-glucuronosyltransferases [4]. UGTs play a critical role in the metabolism and clearance of numerous endogenous (e.g., steroid hormones, bile acids, bilirubin, fatty acids) and exogenous (dietary constituents, environmental toxins and carcinogens, therapeutic drugs) compounds [3,5]. 

The expression profiles of *UGT* genes in human tissues have been investigated at RNA and protein levels using multiple approaches. Due to the lack of specific antibodies for most UGT enzymes, there is the only analysis of protein expression in human tissues for a subset of *UGT* genes (e.g., 1A1, 1A6, 2B7, 2B15, 2B17, 2B28) using custom-developed antibodies via immunohistochemistry, immunoblotting, or tissue microarrays [6,7,8,9,10,11,12]. There are commercial UGT antibodies from several companies (e.g, Sigma, Abcam), but their specificities have not yet been vigorously validated and have been reported to recognize several highly homologous UGT enzymes [11,13]. To overcome this limitation, recent studies have used stable isotope-labeled peptide-based liquid chromatography-tandem mass spectrometry (LC-MS/MS) [14,15,16,17,18,19,20]. However, there is more extensive data on UGT mRNA expression in human tissues and cell lines that have been generated using isoform-specific reverse transcriptase quantitative real-time polymerase chain reaction (RT-qPCR) [21,22,23,24,25,26,27] and RNA sequencing technology (RNAseq) [28,29,30]. RNAseq studies are particularly powerful as they provide accurate isoform-specific and high-throughput quantification of UGT transcripts. Collectively, these studies have demonstrated the widespread expression of *UGT* genes in normal human tissues. Tissues involved in detoxification, particularly liver, and to a lesser degree, kidney and gut, express the widest range of UGT isoforms and typically the highest transcript levels. However, a subset of UGTs shows high expression in tissues that are not generally associated with drug metabolism and detoxification. These UGTs may be important for local control of endogenous metabolites (such as steroids) and could, therefore, mediate intra-tissular drug metabolism. 

Several studies have assessed the expression profiles of *UGT* genes and their deregulation in human cancers that are derived from drug-metabolizing tissues/organs, including liver cancer [31,32], kidney cancer [20], colon cancer [7,33,34], and gastric cancers [35,36]. However, little is known about the expression profiles of *UGT* genes and their deregulation in human cancers that are derived from non-drug-metabolizing tissues.

As recently reviewed [37], case-control studies have shown that a large number of genetic polymorphisms of *UGT* genes are associated with cancer development and progression. This is believed to be related to the critical roles of UGT enzymes in the systemic metabolism and clearance of carcinogens, cancer-modulating molecules, and anticancer drugs. The expression and activity of *UGT* genes within the tumor may also impact cancer development and progression through intratumoral inactivation of carcinogens and anticancer drugs. For example, high *UGT2B17* expression has recently been shown to be associated with poor prognosis in chronic lymphocytic leukaemia (CLL), partly due to enhanced local inactivation of anti-leukaemic drugs (e.g., fludarabine) within CLL cells [38,39]. Therefore, it is necessary to systematically assess whether the intratumoral expression of *UGT* genes could be associated with clinical outcomes in different human cancers.

The Cancer Genome Atlas (TCGA) project analysed over 20,000 primary cancer patients from 33 different cancer types and provides freely-accessible databases for genome-wide molecular profiles (e.g., RNAseq datasets) as well as clinicopathological data (e.g., survival times) for cancer patients (https://gdc.cancer.gov, accessed on 20 June 2021) [40]. Using the RNAseq and clinical datasets from the TCGA project, we recently reported the expression profiles of core ADME genes and their association with patient survival in 21 different TCGA cancer types [41]. Through analyses of the RNAseq and clinical datasets from the TCGA project, the present study defines the expression profiles of all *UGT* genes in 33 different TCGA cancer types and reports a subset of *UGT* genes that are significantly associated with survival rates in specific cancers. We further identify the *UGT* genes that are deregulated in 12 cancer types.

## 2. Materials and Methods

### 2.1. Assessment of the Expression Profiles of UGT Genes in Human Cancers 

The expression profiles of *UGT* genes in cancers were assessed using RNAseq data of 9514 tumor samples from 33 different TCGA cancer types [42] (Table 1). RSEM is one of the most frequently used methods for quantifying transcript abundances from RNASeq data [43]. The mRNA levels (RNASeqV2) of *UGT* genes in 5 TCGA cancers (ESCA, LAML, STAD, OV, UCEC) were obtained as normalized RSEM values from the TCGA database Firehose (http://gdac.broadinstitute.org/, accessed on 20 June 2021; data and analysis version: 2016_01_28). The normalized RSEM values of *UGT* genes for the remaining 28 TCGA cancer types were obtained from the PanCanAtlas database (EBPlusPlusAdjustPANCAN_IlluminaHiSeq_RNASeqV2.geneExp.tsv, accessed on 20 June 2021) (https://gdc.cancer.gov/about-data/publications/pancanatlas, accessed on 20 June 2021).

There are 22 functional *UGT* genes in the human genome [44]. The RSEM values of 2 *UGT* genes (*UGT2B17*, *UGT2A2*) were not available from the Firehose and PanCanAtlas databases, and thus, these 2 *UGT* genes were excluded from our analysis in this study. The normalized RSEM values of the assessed 20 *UGT* genes in 9514 tumor samples are provided in Table S1. The expression levels (e.g., medians, means) of each *UGT* gene in each of the 33 different cancer types were calculated using GraphPad Prism (version 8.1.2, GraphPad Software Inc, San Diego, CA, USA) and are presented in Table S2. For simplicity, we defined high, low, and no expression of a *UGT* gene by a median normalized RSEM level of >32, between 1–32, and <1, respectively. The variable expression of *UGT* genes within and between cancers are presented using the box-and-whisker plots (Figures S1–S4). 

### 2.2. Assessment of the Deregulation of UGT Genes in Human Cancers

Of the 33 TCGA cancer datasets, 12 had at least 20 patients with RNAseq data (RNAseqV2) available for both tumor tissues and matched adjacent non-cancerous tissues (Table 1). Using RNAseq data from these patients, we assessed whether *UGT* genes were differentially expressed in cancer tissues as compared to matched adjacent non-cancerous tissues. Briefly, the RNAseq data (RNASeqV2) was downloaded as high-throughput sequencing (HT-seq) counts from the TCGA legacy database (the human genome hg19 reference assembly) using the “TCGAbiolinks” R package (https://bioconductor.org/packages/release/bioc/html/TCGAbiolinks.html, accessed on 10 May 2021). To remove the distributional differences between within-lane and between-lane read counts, the EDAseq package in R (https://bioconductor.org/packages/release/bioc/html/EDASeq.html, accessed on 10 May 2021) was used, and genes were filtered with a quantile threshold of 0.25 [45]. Table S3 provides HT-seq counts for *UGT* genes in matched tumor and adjacent non-cancerous tissues for 611 patients from the assessed 12 TCGA cancer types. 

The differential gene expression analysis between matched cancerous and adjacent non-cancerous tissues were assessed using the DESeq2 program as recently reported [31]. DESeq2 is one of the most frequently used programs for differential gene expression analysis [46]. Briefly, DESeq2 uses a Wald test for statistically significant testing. The Wald test *p*-values from the genes that pass the independent filtering step are adjusted for multiple testings using the Benjamini–Hochberg test. An adjusted *p*-value of <0.01 was considered statistically significant. DESeq2 reports a log2FoldChange for the differential expression of each gene and the associated Standard Error for the log2FoldChange estimate (lfcSE). Differentially expressed genes were defined by a log2FoldChange of >2 (equivalent to an upregulation of >4-fold) or <−2 (equivalent to downregulation of >4-fold). Table S4 shows the results from DESeq2 analysis pertaining to *UGT* genes. 

Oncomine is a publicly accessible database that analysed hundreds of whole-genome gene expression datasets from normal and cancer tissues (www.oncomine.org, accessed on 10 May 2021) [47]. Using this platform, we further analysed six non-TCGA cancer datasets to verify our findings from TCGA cancer types: prostate cancer [48], lung cancer [49,50], colon cancer [51], gastric cancer [52], and kidney cancer [53]. All of these studies quantified whole-genome gene expression profiles using DNA microarrays, including Affymetrix U133plus 2.0 arrays [49,50,51], Affymetrix “U95a” arrays [48], Affymetrix HG-U133A arrays [53], or custom-made cDNA microarrays containing 44,500 cDNA clones, representing 30,300 genes [52].

### 2.3. Assessment of Associations between the Intratumoral Expression Levels of UGT Genes and Overall Survival of Cancer Patients Using Kaplan–Meier Survival Analysis

The TCGA Pan-Cancer Clinical Data Resources (TCGA-CDR) demonstrated the values of the clinical survival data of 33 TCGA cancer types for reliable survival analyses [42]. The TCGA-CDR collected overall survival data and other clinicopathological parameters for 11,160 patients from 33 different TCGA cancer types. We downloaded these survival data (i.e., TCGA-CDR-Supplemental Table S1) from the PanCanAtlas database (https://gdc.cancer.gov/about-data/publications/pancanatlas, accessed on 4 June 2021). Of these patients, only 9514 patients with RNAseq data (normalized RSEM values as described above) available for tumour samples were included in our survival analyses (Table 1 and Table S1). 

Overall survival (OS) time was defined as the time from the day at diagnosis to the date of death (dead patients) or the date of the last follow-up (censored patients). The Kaplan–Meier survival analysis is a common approach for clinical survival analysis [54]. Using GraphPad Prism (version 8.1.2), we performed Kaplan–Meier plots and logrank tests to assess the potential associations between intratumoral mRNA levels (normalized RSEM values) of *UGT* genes and OS rates for each of the 33 TCGA cancer types. For *UGT* genes that were expressed in over 50% of the tumor samples, we separated the patients by gene expression into a high-expression group (upper 50 percentile) and low/no-expression group (lower 50 percentile) and performed logrank tests. For *UGT* genes that were expressed in 10–50% of the tumor samples, we separated the patients by gene expression into expression group and no-expression group and performed logrank tests. *UGT* genes that were expressed in less than 10% of the tumor samples were excluded from survival analysis. As a varying number of *UGT* genes were expressed in different cancer types (Table S1), the number of independent logrank tests performed varied among different cancers, ranging from 20 tests in LIHC to 3 tests in UVM. To control false-positive discovery rates, we adjusted the logrank *p*-values for each cancer type using Bonferroni correction, the most stringent test for multiple testing correction as recently reported [41]. A Bonferroni-corrected cutoff logrank *p*-value of <0.05 was considered statistically significant. Table S2 lists both logrank and Bonferroni-corrected *p*-values and the associated hazard ratios (HR) and 95% confidence intervals (CI) for all independent logrank tests that assessed the potential associations between intratumoral expression levels of *UGT* genes and overall survival rates in 33 different TCGA cancer types. 

## 3. Results

### 3.1. The Expression Profiles of UGT Genes in Human Cancers

**Figure 1:**
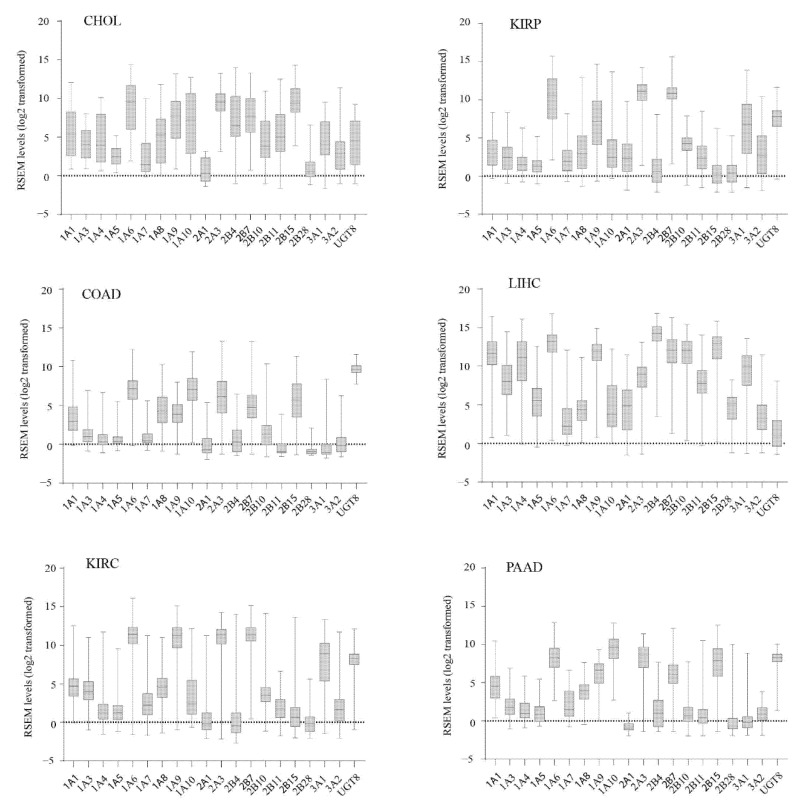
The expression profiles of *UGT* genes in six different types of TCGA cancers. The log2-transformed expression levels (RSEM values) of *UGT* genes in six TCGA cancer types (CHOL, COAD, KIRC, KIRP, LIHC, PAAD) are presented using the box-and-whisker plots that show the distribution of the expression levels (minimum, first quartile, median, third quartile, and maximum) in each of these cancers.

We examined the expression profiles of *UGT* genes in 9514 tumors of 33 different cancer types using RNAseq data from the TCGA project. Table 1 lists the number of patients for each cancer type that was analysed in this study. The distribution of *UGT* expression levels within each cohort are represented using box-and-whisker plots; however, to simplify the discussion, we also defined high, moderate, and low expression criteria (see Methods). Overall, a unique set of *UGT* genes was expressed in different cancers (Figure 1, Figures S1 and S2). LIHC (Liver Hepatocellular Carcinoma) showed high level expression of the broadest range of *UGT* genes (12 genes: *1A1*, *1A3*, *1A4*, *1A6*, *1A9*, *2A3*, *2B4*, *2B7*, *2B10*, *2B11*, *2B15*, *3A1*) (Figure 1), which is consistent with the abundant expression of these *UGT*s in normal liver tissues [44]. Five other cancer types also highly expressed at least 6 *UGT* genes: 1) CHOL (*1A1*, *1A6*, *1A9*, *1A10*, *2A3*, *2B4*, *2B7*, *2B15*), 2) COAD (*1A6*, *1A9*, *2A3*, *2B7*, *2B15*, *UGT8*), 3) KIRC (*1A6*, *1A9*, *2A3*, *2B7*, *3A1*, *UGT8*), 4) KIRP (*1A6*, *1A8*, *2A3*, *2B7*, *3A1*, *UGT8*) and 5) PAAD (*1A6*, *1A10*, *2A3*, *2B7*, *2B15*, *UGT8*) (Figure 1). However, eight cancer types (ACC, BRCA, LAML, MESO, PCPG, PRAD, SARC, UVM) did not show high expression of any *UGT* gene (Figure S2).

**Figure 2:**
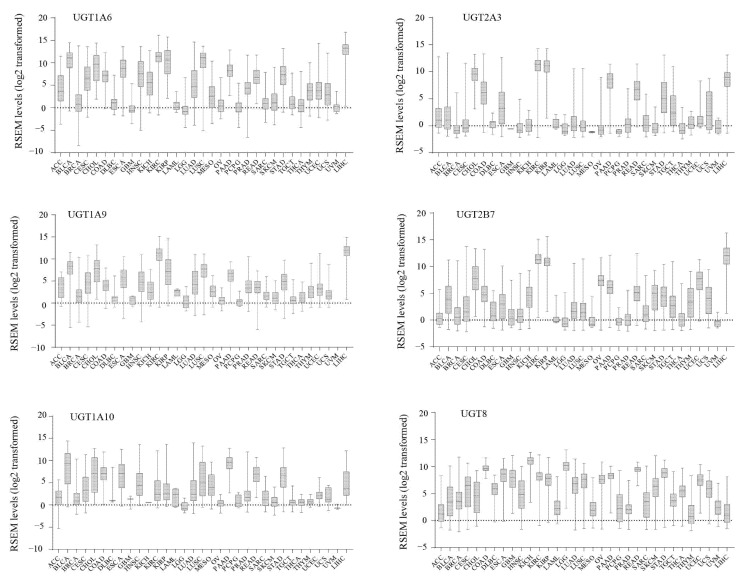
The expression profiles of 6 *UGT* genes (*1A6*, *1A9*, *1A10*, *2A3*, *2B7*, *UGT8*) in 33 different types of TCGA cancers. The log2-transformed expression levels (RSEM values) of 6 *UGT* genes in 33 TCGA cancer types as indicated at the X-Axis are presented using the box-and-whisker plots that show the distribution of the expression levels (minimum, first quartile, median, third quartile, and maximum) in each of these cancers.

Of the 20 *UGT* genes assessed, 6 (*1A6*, *1A9*, *1A10*, *2A3*, *2B7*, *UGT8*) showed high expression in at least 5 different cancer types. The findings for these 6 genes are presented in Figure 2. Expression profiles of the remaining *UGTs* are provided in Figures S3 and S4. *UGT8* was the most broadly expressed and abundant *UGT*, showing high expression in 18 cancers (CESC, COAD, DLBC, ESCA, GBM, KICH, KIRC, KIRP, LGG, LUAD, LUSC, OV, PAAD, READ, SKCM, STAD, UCEC, UCS) (Figure 2) that are derived from a wide range of tissues including the gut, brain, kidney, lung, ovary, uterus, pancreas, skin and lymphoid system; however, *UGT8* was low/absent in LIHC. This pattern is consistent with its high expression in a broad range of normal human tissues but lack of expression in the liver [44]. *UGT1A6* showed high expression in 13 cancer types (BLCA, CESC, CHOL, COAD, ESCA, HNSC, KIRC, KIRP, LIHC, LUSC, PAAD, READ, STAD) (Figure 2) that are derived from a variety of tissues, including many drug-metabolizing tissues (e.g., liver, kidney, gut). However, 8 *UGT* genes (*1A3*, *1A4*, *1A5*, *2A1*, *2B4*, *2B10*, *2B11*, *2B28*) were highly expressed in LIHC, but none of them showed high expression in any of the remaining 32 cancer types (Figure S3). Consistent with their extrahepatic expression profiles in normal tissues, 3 *UGT1As* (*1A7*, *1A8*, *1A10*) had low expression in LIHC, but they were moderately/highly expressed in several non-hepatic cancer types (Figure S3). In particular, *UGT1A10*, which is generally highly expressed in the gut [44], showed high expression in 8 cancer types, including those spanning the whole proximal-distal gut axis (BLCA, CHOL, COAD, ESCA, LUSC, PAAD, READ, STAD) (Figure 2). Expression of *UGT3A1* was high in cancers associated with the hepatobiliary and renal excretion systems (CHOL, KIRC, KIRP, LIHC). This pattern was distinct from that of *UGT3A2*, which was only highly expressed in cancers derived from the uterus and testis (TGTC, UCEC, UCS) (Figure S4). These 2 *UGT3A* genes had low/no expression in all other cancer types.

### 3.2. Inter-Individual Variation in UGT Expression within Human Cancers

The expression of *UGT* genes is highly dynamic and influenced by multiple extrinsic stimuli and signaling pathways; as such, many *UGTs* show exceptionally wide interindividual variation in normal tissues, particularly in the liver [3,44]. Thus, it was of interest to determine the inter-individual variability of *UGT* expression within cancer cohorts. The variable expression of *UGT* genes within 33 different cancer types are presented using the box-and-whisker plots that show the distribution of the expression levels (minimum, first quartile, median, third quartile, and maximum) in each of these cancers (Figure 2, Figures S3 and S4). Overall, all *UGT* genes showed inter-individual variable expression in each of the 33 cancer types. For example, *UGT1A1* was detected in 362 of the 365 LIHC tumors with RSEM ranging from 1.6 to 87,075. A total of 82 tumours had an expression level of >10,000, whereas 88 tumours had an expression level of <1000 (Table S1). This high interindividual variable expression for *UGT1A1* and other *UGT* genes may result in intratumoral variabilities in the conjugation of UGT substrates and hence impact cancer progression and patient survival as assessed in Section 3.4.

### 3.3. Deregulation of UGT Genes in Human Cancers

To examine the deregulation of *UGT* genes in cancers, we compared the expression levels of *UGT* genes between matched cancerous and adjacent non-cancerous tissues in 611 patients from 12 different cancer types where these data were available (Tables S3 and S4). Among the 20 *UGT* genes assessed, only 4 (*1A4*, *1A5*, *2B10*, *2B28*) showed no deregulation in any cancer. The other 16 *UGT* genes were either upregulated or downregulated in at least 1 cancer type (Table 2). Among these genes, 8 were upregulated (*1A3*, *1A6*, *1A7*, *UGT8*) or downregulated (*1A8*, *2A3*, *3A2*, *2B7*) consistently in at least 2 cancer types, and 5 genes (1A1, 1A9, 1A10, 2A1, 2B15) showed upregulation in some cancers but downregulation in other cancer types. For example, *UGT2A1* was downregulated in 4 cancers (HNSC, KICH, KIRC, KIRP) but upregulated in LUSC. Of the 12 *UGT* genes that were highly expressed in LIHC (described above), only *UGT2B11* showed downregulation in this cancer. Consistent with its low expression in the normal liver [44], *UGT1A10* was barely expressed in non-cancerous liver tissues; however, it was significantly upregulated in LIHC (Figure S5).

**Figure 3:**
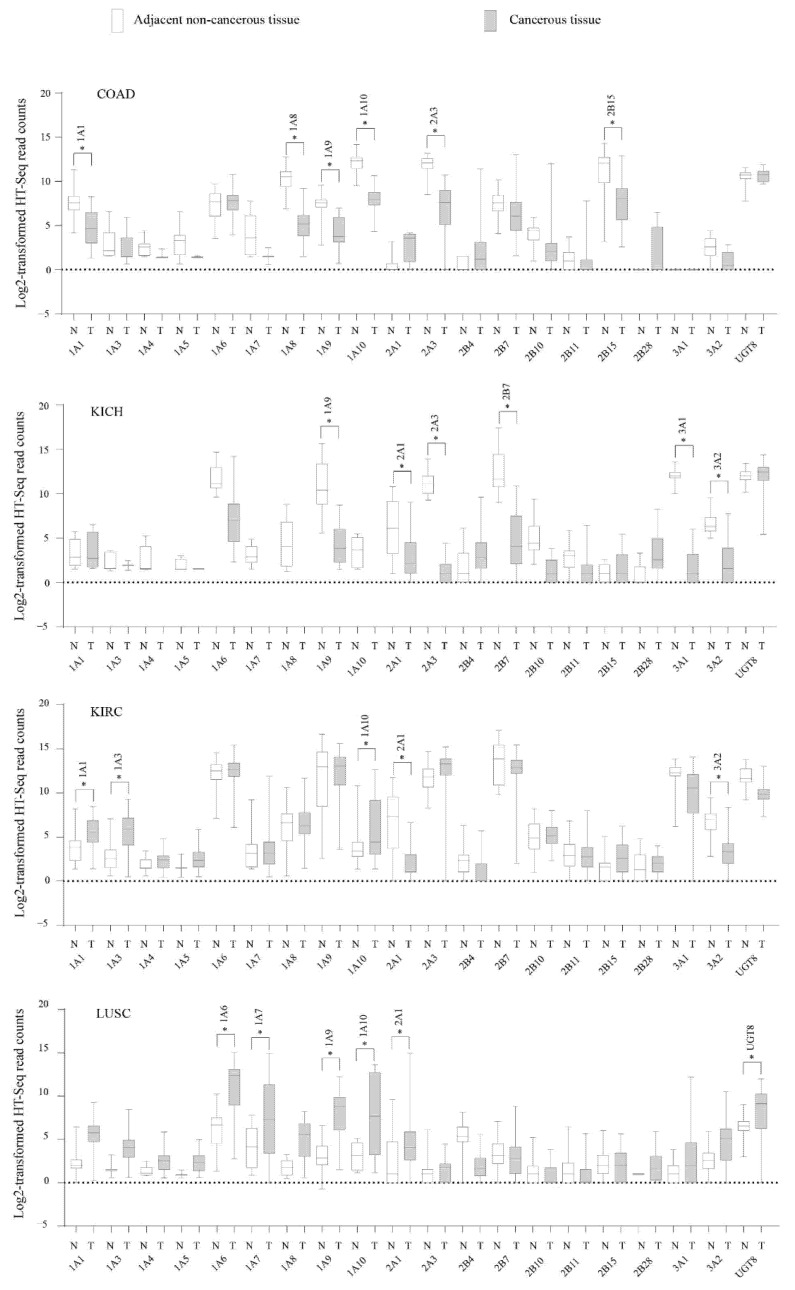
Expression of *UGT* genes in matched cancerous (T) and adjacent non-cancerous (N) tissues from four TCGA cancer types (COAD, KICH, KIRC, LUSC). The log2-transformed expression levels (HT-Seq counts) of *UGT* genes in matched cancerous (T) and non-cancerous (N) tissues from four TCGA cancer types as indicated are presented using the box-and-whisker plots that show the distribution of the expression levels (minimum, first quartile, median, third quartile, and maximum) in each of these cancers. Up/downregulated *UGT* genes are highlighted with * indicating an adjusted *p*-value of <0.01.

Using the Oncomine platform as described in detail in Section 2, we analysed six non-TCGA cancer datasets that reported genome-wide differential gene expression analysis using DNA microarrays. Our results showed consistent findings for nearly half (15/37) of the significant gene deregulation patterns identified in the TCGA cancer datasets (Table 2). In TCGA COAD, 6 *UGT* genes (*1A1*, *1A8*, *1A9*, *1A10*, *2A3*, *2B15*) were downregulated (Figure 3); all of these genes except *UGT1A10* were also downregulated in the Kaiser colon cancer dataset [51] (Table 2). In TCGA LUSC, 6 *UGT* genes were upregulated (Figure 3), 3 of these genes (*1A6*, *1A9*, *UGT8*) were also upregulated in the Hou lung cancer dataset (Table 2) [50]. In TCGA KICH, 6 *UGT* genes (*1A9*, *2A1*, *2A3*, *2B7*, *3A1*, *3A2*) were downregulated (Figure 3); 2 of which (2A1, 2A3) were also downregulated in the Jones renal cancer dataset (Table 2) [53]. In TCGA KIRC, 3 *UGT* genes (*1A1*, *1A3*, *1A10*) were upregulated and 2 *UGT* genes (*2A1*, *3A2*) were downregulated (Figure 3). The downregulation of *UGT2A1* was also seen in the Jones Renal cancer dataset [53] (Table 2). 

### 3.4. Associations between Intratumoral UGT Expression Levels and Overall Survival of Cancer Patients

**Figure 4:**
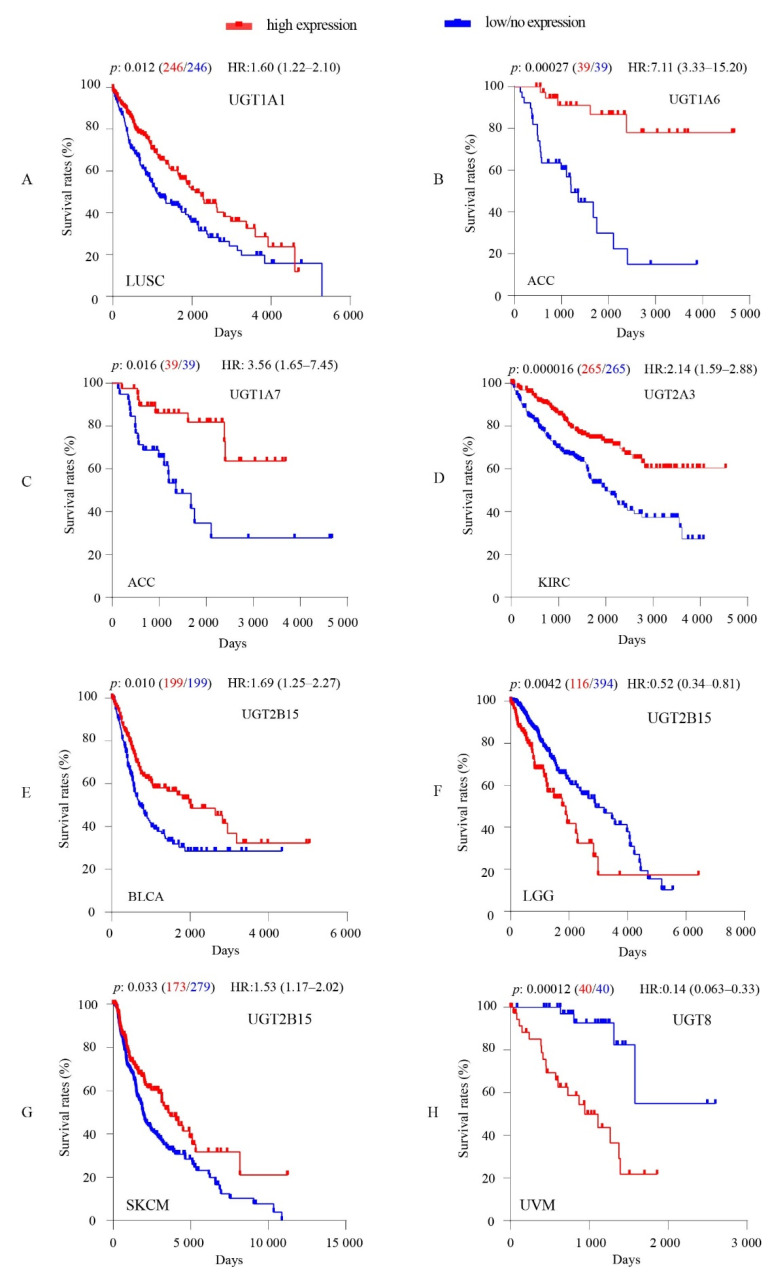
Kaplan–Meier plots and logrank tests show significant associations of intratumoral expression levels of *UGT* genes with overall survival rates in TCGA cancer types as indicated. For Kaplan–Meier survival analysis, the patients were separated into high-expression groups and low/no-expression group by gene expression levels as described in the Materials and Methods Section. The number of patients in each group for each Kaplan–Meier plot/logrank test was given in bracket following the *p*-value. Hazard Ratios (HR) and 95% confidence intervals (95% CI) are also shown. Logrank *p*-values were adjusted using Bonferroni correction as described in the Materials and Method Section. A Bonferroni-corrected cutoff logrank *p*-value of <0.05 indicates statistical significance.

Using Kaplan–Meier plots and logrank tests followed by Bonferroni multiple corrections, we assessed the potential association of the intratumoral expression levels of *UGT* genes with overall survival (OS) rates in each of the 33 TCGA cancer types (Table S2). Overall, our results showed that the expression levels of 6 *UGT* genes (*1A1*, *1A6*, *1A7*, *2A3*, *2B15*, *UGT8*) were significantly associated with OS rates in at least one cancer type (Figure 4). Of these genes, four showed significant association with increased OS rates (favourable survival), namely (1) *UGT1A1* in LUSC (Figure 4A), (2) *UGT1A6* in ACC (Figure 4B), (3) *UGT1A7* in ACC (Figure 4C), and (4) *UGT2A3* in KIRC (Figure 4D). *UGT8* was significantly associated with decreased OS rates (unfavourable survival) in UVM (Figure 4H). *UGT2B15* showed significant association with increased OS rates in BLCA and SKCM) (Figure 4E,G) but decreased OS rates in LGG (Figure 4F).

## 4. Discussion

We recently analysed the RNAseq data from the Human Protein Atlas [55] (https://www.proteinatlas.org, accessed on 10 January 2019) and the Genotype-Tissue Expression (GTEx) project [56] and reported the expression profiles of all 22 *UGT* genes in a panel of 43 normal human tissues [44]. This demonstrated that many *UGT* genes that have been traditionally associated with liver or other detoxifying tissues, in fact, have complex body-wide expression patterns. As an example, seven UGTs (*1A1*, *1A6*, *1A10*, *2B7*, *2B15*, *2B17*, and *UGT8)* showed expression in more than 20 different tissues [44]. This widespread expression supports an important role for UGTs in intra-tissular drug/xenobiotic exposure, as well as controlling endogenous lipophilic metabolites and signaling molecules. These roles may also contribute to the systemic clearance of UGT substrates. UGT expression within tumors derived from various tissues could similarly affect intratumoral drug exposure, as well as levels of endogenous growth-regulatory chemicals. In the present study, we analysed the RNAseq data of 9514 cancer patients from the TCGA project, allowing us to characterize the expression profiles of 20 *UGT* genes in 33 different cancer types. 

Overall, *UGT* genes exhibited cancer-specific expression profiles and high interindividual variabilities within cancer cohorts. Four (*1A6*, *1A10*, *2B7*, *UGT8*) of the aforementioned seven *UGT* genes that are widely expressed in normal tissues were also highly expressed in more than five different cancer types (Figure 2). Notably, *UGT8* and *UGT1A6* showed high expression in 18 and 13 different cancer types, respectively. A total of 25 cancer types (BLCA, CESC, CHOL, COAD, DLBC, ESCA, GBM, HNSC, KICH, KIRC, KIRP, LGG, LIHC, LUAD, LUSC, OV, PAAD, READ, SKCM, STAD, TGCT, STAD, THCA, THYM, UCS) highly expressed at least 1 *UGT* gene. Previous studies have shown high expression of *UGT* genes in drug-metabolizing tissues (e.g., liver, kidney, colon) [20,22,23,27,30,33]. Consistently, we found abundant expression of similar *UGT* genes in cancers that are derived from drug-metabolizing tissues (LIHC, KIRC/KIRP, COAD) (Figure 1). For example, 12 *UGT* genes were abundantly expressed in liver cancer (LIHC) in accordance with their high expression in the normal liver. The present study represents the first to comprehensively assess the expression profiles of *UGT* genes in nearly 30 different cancers derived from non-drug-metabolizing tissues. Collectively, our results demonstrate widespread expression profiles of *UGT* genes in human cancers, implying active metabolism of UGT substrates within the tumors that are derived from not only drug-metabolizing tissues but also non-drug-metabolizing tissues. 

Deregulation of *UGT* genes have been previously reported in cancers that are derived from drug-metabolizing organs (LIHC [31,32], KIRC [20], COAD [7,33,34], STAD [35,36]). In the present study, we compared the expression of *UGT* genes between matched cancerous and non-cancerous tissues of 611 TCGA patients in 6 cancers derived from drug-metabolizing tissues (COAD, KICH, KIRC, KIRP, LIHC, STAD) and another six cancers derived from non-drug-metabolizing tissues (BRCA, HNSC, LUAD, LUSC, PRAD, THCA). Overall, we found 16 *UGT* genes (*1A1*, *1A3*, *1A6*, *1A7*, *1A8*, *1A9*, *1A10*, *2A1*, *2A3*, *2B4*, *2B7*, *2B11*, *2B15*, *3A1*, *3A2*, *8*) that were up/down-regulated in at least 1 cancer type (Table 2). Nearly half of these up/downregulated *UGT* genes were consistent with the observations from the aforementioned studies or corroborated by other microarray-based gene expression profiling studies [48,49,50,51,52,53]. UGT enzymes are involved in the inactivation of procarcinogens and carcinogens, and hence their downregulation may represent a putative early event in carcinogenesis as previously suggested [7,32,33,35]. It is also possible that *UGT* down-regulation may be a late event linked to cellular de-differentiation in advanced cancers. Further analysis of UGT expression by cancer stage may help to tease out such possibilities. On the other hand, upregulation of *UGT* genes may be related to anticancer drug resistance, as discussed below.

Given that intratumoural *UGT* expression could enhance the inactivation of anticancer drugs and cancer-growth modulating molecules (e.g., steroids, lipids, fatty acids), it may be predicted to impact cancer progression and patient survival. Indeed, clinical and pre-clinical studies have shown that overexpression of specific *UGT*s can promote resistance to anticancer drugs that they metabolize [57,58,59,60,61,62,63]. In the present study, Kaplan–Meier survival analysis of 9145 patients from 33 different TCGA cancer types identified significant associations of intratumoral expression levels of *UGT* genes with overall survival rates in seven different cancer types, namely LUSC (*1A1*), ACC (*1A6*, *1A7*), BLCA (*2B15*), KIRC (*2A3*), LGG (*2B15*), SKCM (*2B15*), and UVM (*UGT8*) (Figure 4). Of note, the associations of *UGT1A1, UGT2B15,* and *UGT2A3* with favourable OS rates in LUSC [41], BLCA [41], and KIRC [55], respectively, were recently reported. As discussed in detail below, the inactivation of anticancer drugs within the tumor through the UGT conjugation pathway can reduce therapeutic efficacy and patient survival. Analysis of drug regimens received by TCGA patients could test this hypothesis. Therefore, we obtained drug regimen data for these seven TCGA cancer types from NCI Genomic Data Commons (https://gdc.cancer.gov/, accessed on 10 June 2021) (Table S5). Unfortunately, because drug regimens were only available for a small proportion of patients for these seven cancer types, we were not able to assess whether the observed association of UGTs with OS rates could be related to intratumoral inactivation of anticancer drugs. 

Interestingly, our data show that high UGT levels were frequently correlated with increased OS rates (Figure 4). These findings are consistent with recent reports for other drug-metabolizing enzymes (e.g., CYPs, NAT1) [41], and support a hypothesis that intratumoral expression of UGTs and other drug-metabolizing enzymes can impact cancer patient survival through not only drug metabolism but also metabolism of numerous endogenous bioactive molecules (e.g., steroid hormones, amino acids, fatty acids, bile acids) that can modulate cancer growth as briefly discussed below.

Androgens are known to promote bladder cancer (BLCA) development and progression [64,65,66]. The two most potent natural androgens (testosterone and dihydrotestosterone) are primarily inactivated by UGT2B15 and UGT2B17 [67]. Our observed association of high UGT2B15 levels with increased OS rates in BLCA (Figure 4) might be attributable to its intratumoral glucuronidation of androgens within the tumour as recently suggested [41]. Unfortunately, *UGT2B17* data were not available for analysis within the TCGA dataset. We also showed an association of UGT2B15 with increased OS rates in SKCM or decreased OS rates in LGG (Figure 4); however, the underlying mechanisms remains unknown.

Adrenocortical carcinoma (ACC) is a very rare malignancy that originates in the cortex of the adrenal gland, and patients with ACC often have poor clinical outcomes [68]. Surgery remains the only curative treatment for patients with ACC, and mitotane is the most effective drug in adjuvant chemotherapy of ACC or in inoperable ACC [69]. There is no evidence that mitotane and its two activate metabolites [1,1-(o,p’-dichlorodiphenyl)-2,2 dichloroethene (o,p’-DDE), 1,1-(o,p’-dichlorodiphenyl) acetic acid (o,p’-DDA) ] are substrates of any UGT [70]. Some patients with ACC have increased levels of steroid hormones and mineralocorticoids, and they tend to show hypercortisolism and hyperandrogenism [68]. ACC showed moderate expression of *UGT1A6* and *UGT1A7* but low/no expression of other *UGT* genes (Figure S2). Our observed association of high levels of *UGT1A6* or *UGT1A7* with increased OS rates might be related to their intratumoral inactivation and clearance of endogenous bioactive molecules, such as the aforementioned steroid hormones. This hypothesis remains to be investigated. 

We showed a significant association of high UGT8 expression with poor OS rates in Uveal Melanoma (UVM) (Figure 4H). UVM originates from melanocytes within the uveal tract and is the second most common melanoma subtype after cutaneous melanoma [71]. Primary UVM is treated with surgery and radiation; however, remote metastasis (most often in the liver) occurs in nearly 50% of the patients with a very poor prognosis [72]. A 10-gene signature (*SIRT3*, *HMCES*, *SLC44A3*, *TCTN1*, *STPG1*, *POMGNT2*, *RNF208*, *ANXA2P2*, *ULBP1*, *CA12*) that predicts prognosis for this disease has been recently reported [73]. Further studies are warranted to determine whether *UGT8* may be a useful prognostic biomarker for UVM. The mechanism by which *UGT8* could influence UVM is currently speculative. UGT8 galactosidates bile acids [74] and ceramide [75]. The later reaction generates galactosylceramide (GalCer), which can be further converted into sufatide [76]. Ceramides are critical regulators of survival and drug resistance in melanoma, hence UGT8 might control UVM outcomes via modulation of ceramide levels [77]. In support of this notion, UGT8 overexpression was recently shown to promote basal-like breast cancer (BLBC) cell proliferation and invasion through production of GalCer and sufatide [78,79]. Although Cao et al. recently showed an association between high intratumoral UGT8 levels and poor survival in BLBC [79], no association was observed in our analysis of the TCGA BRCA dataset as a whole (1080 patients) or the basal subtype (179 patients) (data not shown). 

The findings of the current study could provide impetus for future translational UGT research. The potential for such translation is supported by numerous clinical and pre-clinical studies. For example, our findings of high interindividual expression variability and deregulation of *UGT* genes within specific cancer types may be relevant to intratumoral exposure of anticancer drugs that are primarily metabolized through UGT conjugation. In support of this idea, pre-clinical and clinical studies have shown that high intratumoral expression of several *UGT* genes (e.g., 1A1, 1A6, *2B7*, *2B17*) contributed to de novo or acquired resistance to various anticancer drugs [39,60,80]. These findings, together with observations in the present study, suggest that assessing intratumoral UGT activity could help to achieve optimal personalized anticancer therapy. Moreover, our observed associations of intratumoral UGT expression with patient survival highlight their potential as prognostic biomarkers. In addition to the data shown here that assessed OS within 33 TCGA cancer types, other studies have reported the prognostic value of various UGTs in specific subsets of cancer patients [6,38,39,75]. For example, previous work shows that UGT2B17 and UGT2B28 are overexpressed in advanced and metastatic prostate cancer and associate with poor outcomes [6,81,82,83]. Pre-clinical studies in mouse xenograft models show that UGT2B17 overexpression promotes androgen-independent tumor progression via a pathway that may involve tyrosine-protein kinase Src [82]. UGT2B17 is also prognostic in chronic lymphocytic leukaemia (CLL), where high expression is associated with shorter treatment-free and overall survival primarily through its intracellular inactivation of anti-leukaemic drugs such as fludarabine [39]. In addition, we have shown that UGT2B15 and UGT2B17 are prognostic in specific molecular subtypes of breast cancer as defined by the METABRIC (Molecular Taxonomy of Breast Cancer International Consortium) project [84]. Based on these previous studies and the new data presented herein, we suggest that future work could focus on developing selected UGTs as clinically actionable biomarkers and/or therapeutic targets for new drug discovery. 

## 5. Conclusions

In conclusion, the present study reported the unique expression profiles of UGT genes in 33 TCGA cancer types and identified the patterns of *UGT* deregulation in 12 TCGA cancer types. We further identified the *UGT* genes whose intratumoral expression was significantly associated with overall survival. Collectively, our results provide compelling evidence for the active metabolism of UGT substrates within tumors, and support further interrogation of *UGT* genes as potential prognostic biomarkers and therapeutic targets. Intratumoral UGT activity can influence cancer progression and patient survival not only through drug metabolism but also through the inactivation of numerous endogenous bioactive molecules that can modulate cancer growth.

## Figures and Tables

**Table 1:** A total of 9514 patients of 33 different types of TCGA cancers were analysed in this study.

Cancer Type	Description	No. of Patients	No. of Paired Cancerous and Non-Cancerous Tissues
ACC	Adrenocortical carcinoma	78	
BLCA	Bladder Urothelial Carcinoma	398	
BRCA	Breast Invasive Carcinoma	1080	113
CESC	Cervical Squamous Cell Carcinoma and		
	endocervical Adenocarcinoma	304	
CHOL	Cholangiocarcinoma	35	
COAD	Colon Adenocarcinoma	235	24
DLBC	Lymphoid Neoplasm Diffuse Large B-cell		
	lymphoma	47	
ESCA	Esophageal Carcinoma	182	
GBM	Glioblastoma Multiforme	148	
HNSC	Head and Neck Squamous Cell Carcinoma	519	43
KICH	Kidney Chromophobe	65	25
KIRC	Kidney Renal Clear Cell Carcinoma	531	72
KIRP	Kidney Renal Papillary Cell Carcinoma	287	32
LAML	Acute Myeloid Leukemia	149	
LGG	Brain Lower Grade Glioma	510	
LIHC	Liver Hepatocellular Carcinoma	365	50
LUAD	Lung Adenocarcinoma	502	58
LUSC	Lung Squamous Cell Carcinoma	492	51
MESO	Mesothelioma	85	
OV	Ovary Serous Cystadenocarcinoma	294	
PCPG	Pheochromocytoma and Paraganglioma	179	
PAAD	Pancreatic Adenocarcinoma	144	
PRAD	Prostate adenocarcinoma	462	52
READ	Rectum Adenocarcinoma	93	
SARC	Sarcoma	258	
SKCM	Skin Cutaneous Melanoma	452	
STAD	Stomach Adenocarcinoma	357	32
TGCT	Testicular Germ Cell Tumors	134	
THCA	Thyroid Carcinoma	504	59
THYM	Thymoma	119	
UCEC	Uterine Corpus Endometrial Carcinoma	370	
UCS	Uterine Carcinosarcoma	56	
UVM	Uveal Melanoma	80	

**Table 2:** *UGT* genes that were up/downregulated in cancerous tissues compared to matched adjacent non-cancerous tissues in TCGA and non-TCGA cancer datasets. A *p*-value of <0.01 is considered statistically significant.

TCGA Datasets				Non-TCGA Datasets		
UGT Genes	Cancer Types	Fold Change	*p*-Values	Fold Change	*p*-Values	Independent Studies
1A1	COAD	−7.0	2.74 × 10^−6^	−10.5	6.66 × 10^−5^	Kaiser S et al., 2007 [51]
1A1	KIRC	4.8	9.69 × 10^−11^			
1A1	STAD	−10.1	1.90 × 10^−5^			
1A3	KIRC	13.4	3.35 × 10^−25^			
1A3	PRAD	6.8	1.49 × 10^7^			
1A6	LUAD	6.5	4.82 × 10^−10^			
1A6	LUSC	48.5	1.63 × 10^−45^	11.9	2.04 × 10^−11^	Hou J et al., 2010 [50]
1A7	LUSC	1074.9	2.96 × 10^−28^			
1A7	STAD	−8.8	0.0051			
1A8	COAD	−27.0	7.70 × 10^−34^	−12.5	2.01 × 10^−5^	Kaiser S et al., 2007 [51]
1A8	HNSC	−6.3	0.00011			
1A9	COAD	−10.8	2.33 × 10^−11^	−11.7	2.66 × 10^−5^	Kaiser S et al., 2007 [51]
1A9	KICH	−229.1	3.36 × 10^−22^			
1A9	LUSC	65.3	3.00 × 10^−33^	14.9	1.19 × 10^−11^	Hou J et al., 2010 [50]
1A10	COAD	−16.5	2.18 × 10^−39^			
1A10	KIRC	39.1	7.84 × 10^−19^			
1A10	LIHC	54.5	4.81 × 10^−12^			
2A1	HNSC	−6.5	0.0033			
2A1	KICH	−8.3	2.24 × 10^−5^	−2.5	1.38 × 10^−4^	Jones J et al., 2005 [53]
2A1	KIRC	−90.5	4.34 × 10^−36^	−4.0	3.63 × 10^−18^	Jones J et al., 2005 [53]
2A1	KIRP	−6.5	0.000673			
2A1	LUSC	16.4	5.19 × 10^−11^			
2A3	COAD	−15.8	9.33 × 10^−22^	−7.6	6.38 × 10^−10^	Kaiser S et al., 2007 [51]
2A3	KICH	−1112.8	2.90 × 10^−101^	−3.1	1.62 × 10^−6^	Jones J et al., 2005 [53]
2B4	PRAD	29.4	2.62 × 10^−25^	4.3	6.18 × 10^−5^	Welsh JB et al., 2001 [48]
2B7	KICH	−110.6	7.06 × 10^−23^			
2B7	KIRP	−6.0	3.99 × 10^−9^			
2B7	STAD	−4.1	0.0093	−2.5	1.15 × 10^−4^	Chen X et al., 2003 [52]
2B7	PRAD	−56.8	4.32 × 10^−18^			
2B11	LIHC	5.3	1.30 × 10^−12^			
2B15	COAD	−10.5	7.09 × 10^−10^	−12.0	3.42 × 10^−6^	Kaiser S et al., 2007 [51]
2B15	LUAD	6	4.56 × 10^−10^	3.3	3.82 × 10^−16^	Okayama H et al., 2003 [49]
3A1	KICH	−661.6	3.10 × 10^−99^			
3A2	KICH	−11.7	2.90 × 10^−8^			
3A2	KIRC	−6.6	3.04 × 10^−22^			
UGT8	LUAD	5.3	1.63 × 10^−26^	3	4.33 × 10^−8^	Hou J et al., 2010 [50]
UGT8	LUSC	6.8	2.45 × 10^−22^	5.8	4.11 × 10^−9^	Hou J et al., 2010 [50]

## Data Availability

For analysis of gene expression profiles, RNASeq data (RSEM) were obtained from the TCGA Firehose database (http://gdac.broadinstitute.org) and the PanCanAtlas database (https://gdc.cancer.gov/about-data/publicatio ns/pancanatlase, accessed on 20 June 2021). For gene differential expression analysis, RNAseq data (HT-seq counts) were obtained from the TCGA legacy database using the “TCGAbiolinks” R package (https://bioconductor.org/packages/release/bioc/html/TCGAbiolinks.html). The survival data of TCGA patients were obtained from the PanCanAtlas database (https://gdc.cancer.gov/about-data/publications/pancanatlas).

## Supplementary Materials

The following are available online at https://www.mdpi.com/article/10.3390/cancers13174491/s1, Figure S1: The expression profiles of *UGT* genes in 10 different types of TCGA cancers; Figure S2: The expression profiles of *UGT* genes in 17 different types of TCGA cancers; Figure S3: The expression profiles of 12 *UGT* genes in 33 different types of TCGA cancers; Figure S4: The expression profiles of *UGT3A* genes in 33 different types of TCGA cancers; Figure S5: Expression of *UGT* genes in matched cancerous (T) and adjacent non-cancerous (N) tissues in 8 different TCGA cancer types; Table S1: The expression levels (normalized RSEM values) of *UGT* genes and clinical datasets of 9514 patients from 33 different types of TCGA cancers; Table S2: The expression levels (means, medians, standard deviations) of *UGT* genes and assessment of their associations with overall survival rates in 33 different TCGA cancer types; Table S3: HT-seq counts for *UGT* genes in matched tumor and adjacent non-cancerous tissues for 611 patients from 12 different TCGA cancer types; Table S4: Differential gene expression analysis of 611 patients from 12 TCGA cancer types using DESeq2 revealed the *UGT* genes that were deregulated in these cancers; Table S5: Drug regimens of 7 TCGA cancer types.
