# Supplementary material for: The Expression Profiles and Deregulation of UDP-Glycosyltransferase (UGT) Genes in Human Cancers and Their Association with Clinical Outcomes
Source: Cancers (Basel). 2021 Sep 6;13(17):4491. doi: 10.3390/cancers13174491 (PMC8430925; doi:10.3390/cancers13174491)
Supplement: Supplementary file 1 [file cancers-13-04491-s001.zip › Supplemental Figures (Figures S1¿CS5).pdf]

cancers

MDPI

Article

The Expression Profiles and Deregulation of UDP-Glycosyltransferase (*UGT*) Genes in Human Cancers and Their Association with Clinical Outcomes

Dong Gui Hu [1,*], Shashikanth Marri [2], Peter I. Mackenzie [1], Julie-Ann Hulin [1], Ross A McKinnon [1] and Robyn Meech [1]

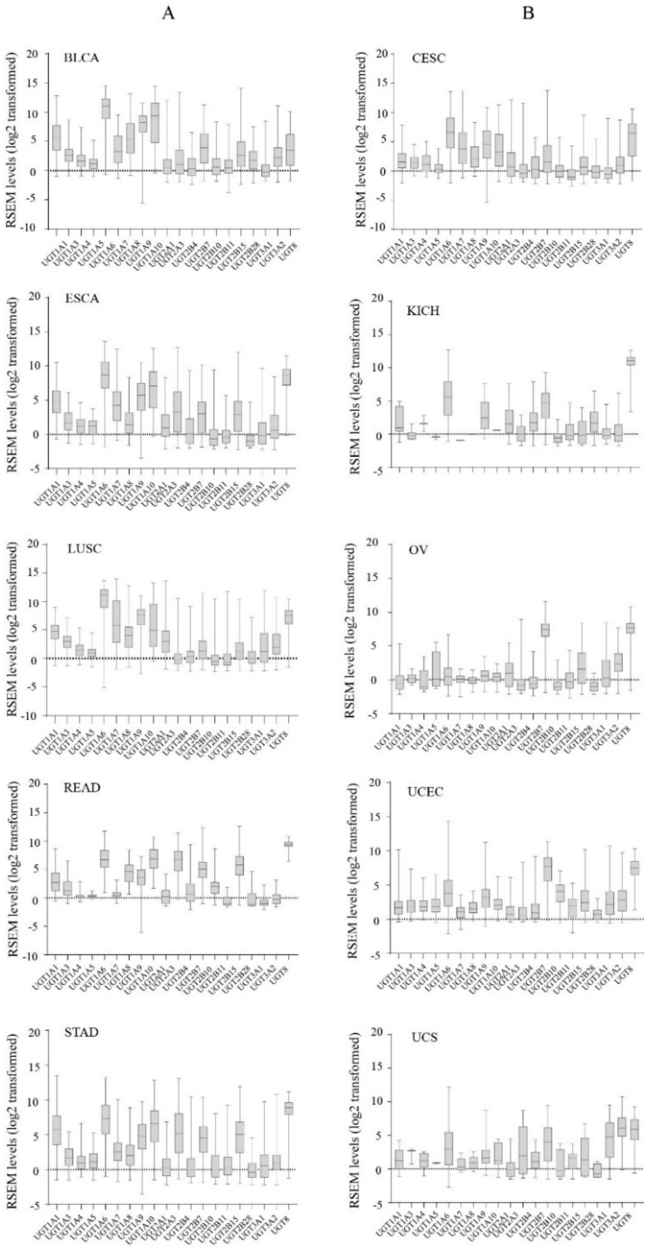

Figure S1. The expression profiles of *UGT* genes in ten different types of TCGA cancers. The log2-transformed expression levels (RSEM values) of *UGT* genes in ten TCGA cancer types as indicated are presented using the box-and-whisker plots that show the distribution of the expression levels (minimum, first quartile, median, third quartile, and maximum) in each of these cancers. Shown are cancers with high expression (log2-transformed value > 5) of 3-5 *UGT* genes (A) or 2 *UGT* genes (B).

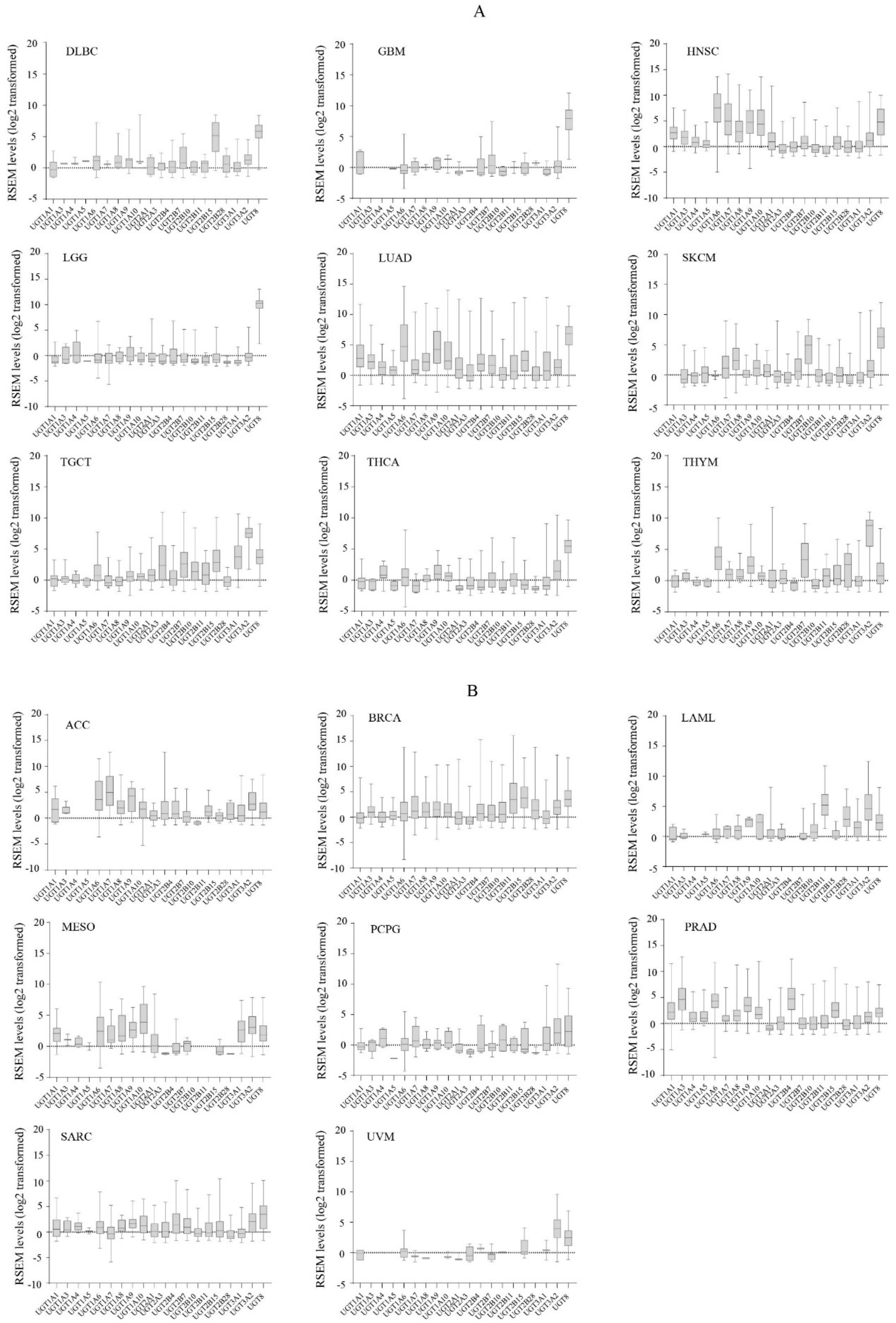

Figure S2. The expression profiles of *UGT* genes in seventeen different types of TCGA cancers. The log2-transformed expression levels (RSEM values) of *UGT* genes in seventeen TCGA cancer types as indicated are presented using the box-and-whisker plots that show the distribution of the expression levels (minimum, first quartile, median, third quartile, and maximum) in each of these cancers. Shown are cancers with high expression (log2-transformed value > 5) of one *UGT* gene (A) or no *UGT* gene (B).

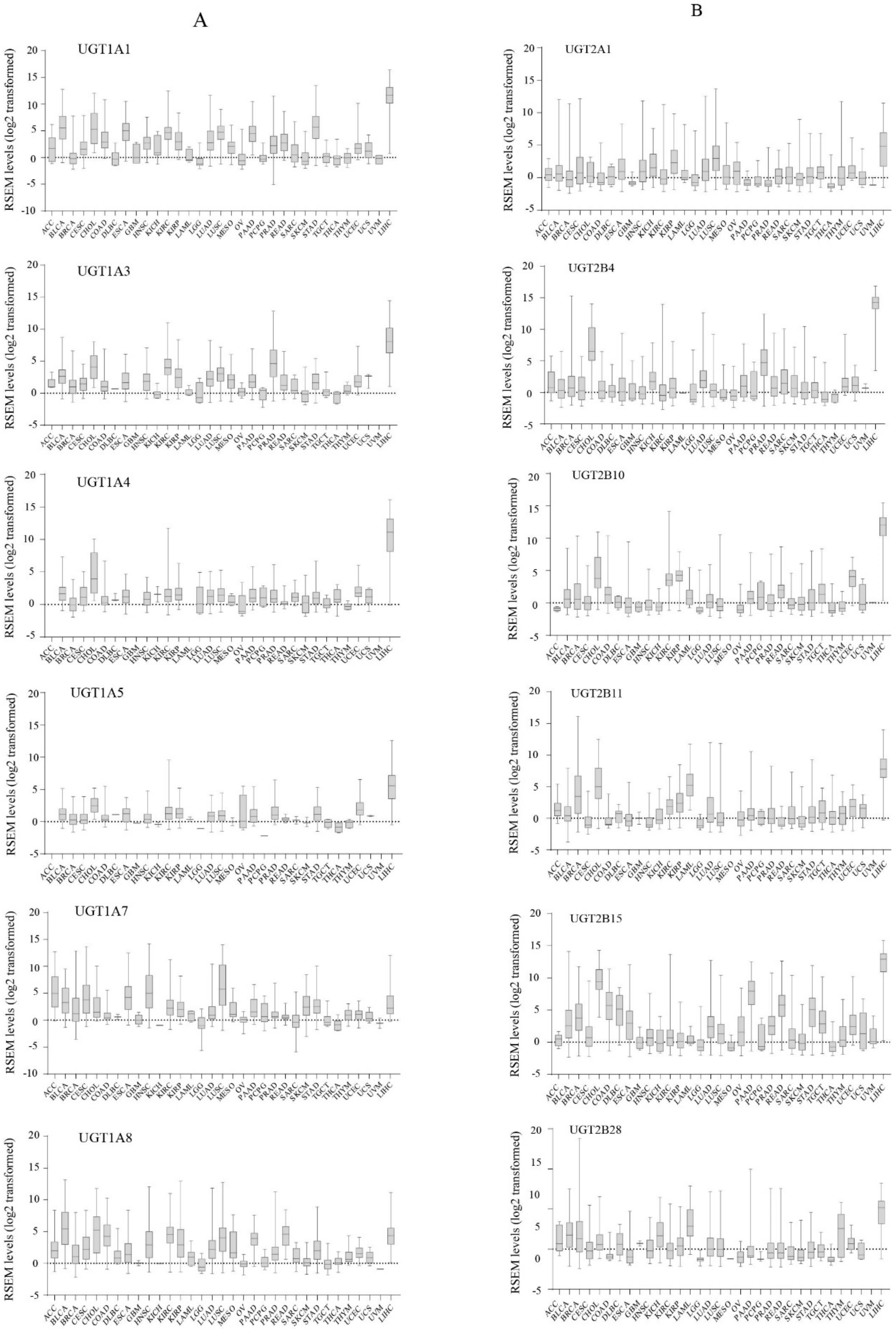

Figure S3. The expression profiles of 12 *UGT* genes in 33 different types of TCGA cancers. The log2-transformed expression levels (RSEM values) of six UGT1A (A) and six UGT2B (B) genes in 33 TCGA cancer types as indicated are presented using the box-and-whisker plots that show the distribution of the expression levels (minimum, first quartile, median, third quartile, and maximum) in each of these cancers.

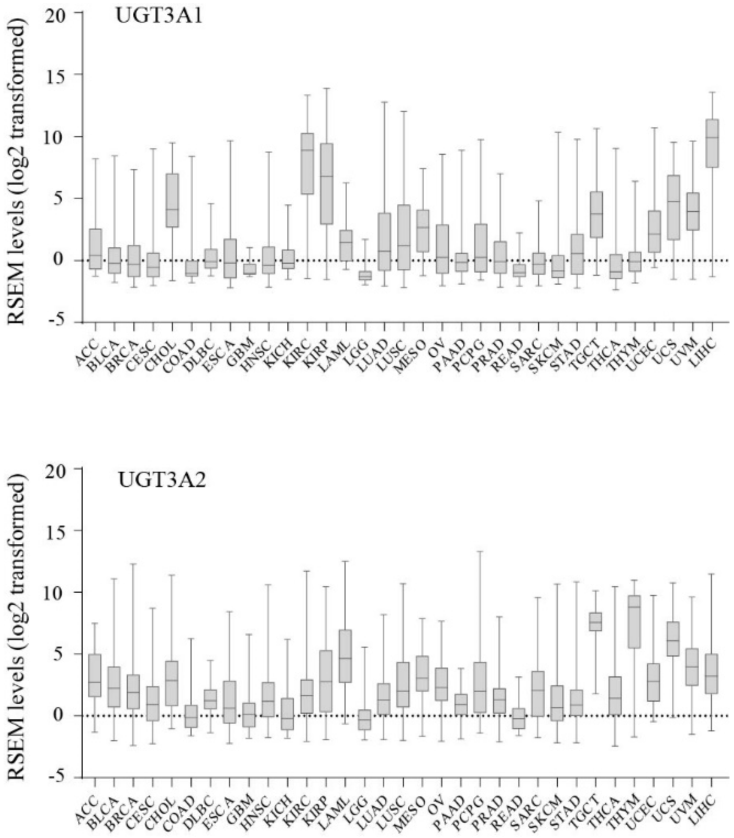

Figure S4. The expression profiles of *UGT3A* genes in 33 different types of TCGA cancers. The log2-transformed expression levels (RSEM values) of *UGT3A1* and *UGT3A2* genes in 33 TCGA cancer types as indicated are presented using the box-and-whisker plots that show the distribution of the expression levels (minimum, first quartile, median, third quartile, and maximum) in each of these cancers.

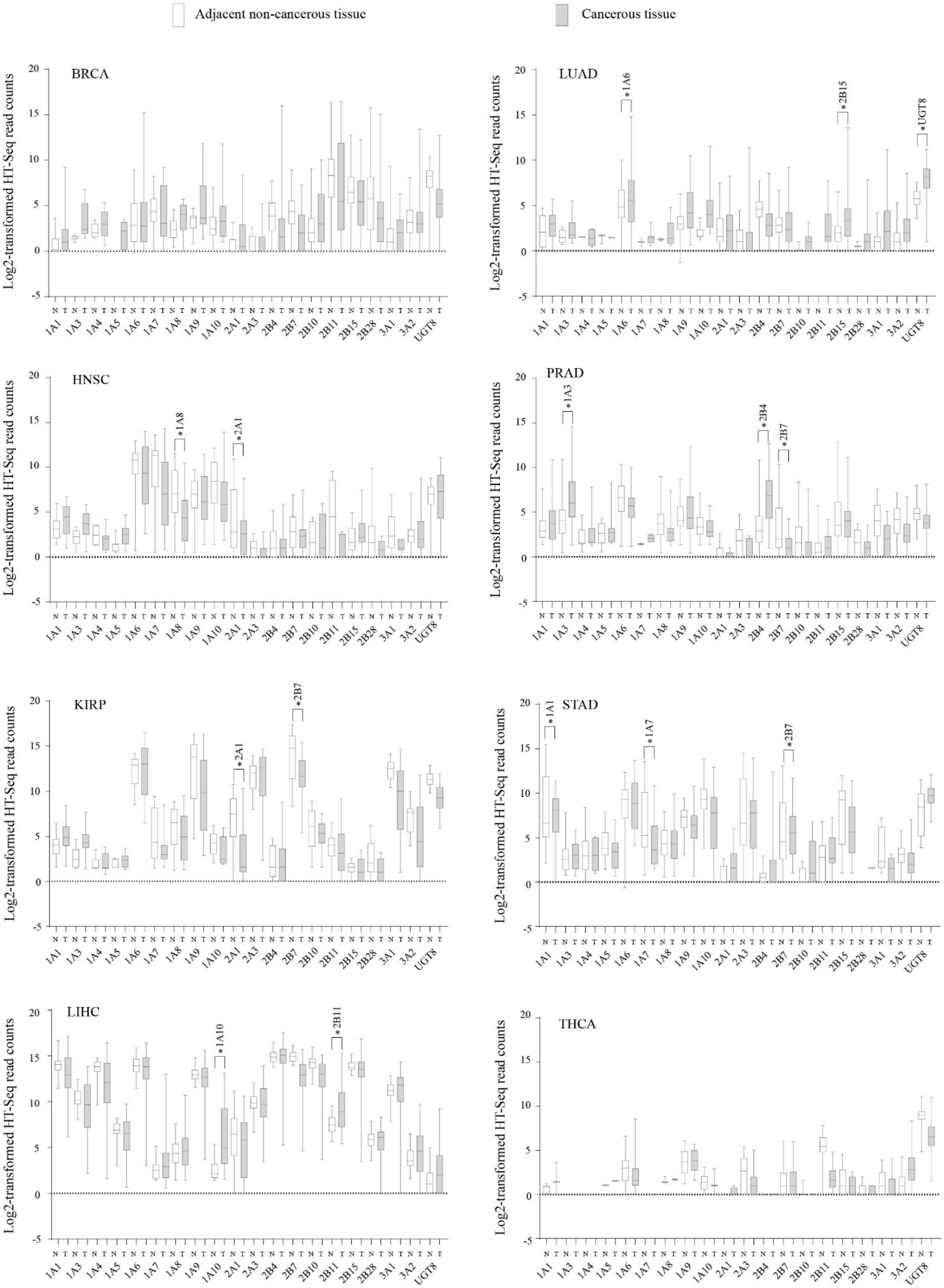

Figure S5. Expression of *UGT* genes in matched cancerous (T) and adjacent non-cancerous (N) tissues in eight different TCGA cancers types. The log2-transformed expression levels (HT-Seq values) of *UGT* genes in matched cancerous (T) and adjacent non-cancerous (N) tissues from eight TCGA cancer types (BRCA, HNSC, KIRP, LIHC, LUAD, PRAD, STAD, THCA) as indicated are presented using the box-and-whisker plots that show the distribution of the expression levels (minimum, first quartile, median, third quartile, and maximum) in each of these cancers. Up/downregulated *UGT* genes are highlighted with * indicating a Benjamini-Hochberg-adjusted p value of < 0.01. .
